# Dynamic reorganization of the actin cytoskeleton

**DOI:** 10.12688/f1000research.6374.1

**Published:** 2015-10-01

**Authors:** Gaëlle Letort, Hajer Ennomani, Laurène Gressin, Manuel Théry, Laurent Blanchoin

**Affiliations:** 1 Laboratoire de Physiologie Cellulaire et Végétale, Institut de Recherches en Technologies et Sciences pour le Vivant, iRTSV, CNRS/CEA/UGA, Grenoble, France; 2 Unité de Thérapie Cellulaire, Hôpital Saint Louis, Institut Universitaire d’Hematologie, UMRS1160, INSERM/AP-HP/Université Paris Diderot, Paris, France

**Keywords:** Actin, Cytoskeleton, Polarization

## Abstract

Cellular processes, including morphogenesis, polarization, and motility, rely on a variety of actin-based structures. Although the biochemical composition and filament organization of these structures are different, they often emerge from a common origin. This is possible because the actin structures are highly dynamic. Indeed, they assemble, grow, and disassemble in a time scale of a second to a minute. Therefore, the reorganization of a given actin structure can promote the formation of another. Here, we discuss such transitions and illustrate them with computer simulations.

## Introduction

Cellular actin assembly can generate a variety of architectures. These highly dynamic actin-based structures lie at the heart of a diverse array of cellular processes ^1, 2^. Actin filaments are found inside cells in three basic patterns: branched filament networks, parallel-, or mixed-polarity filament bundle arrays. These different types of organization can contribute to more complex structures and determine their functions ^3^. Although most of the time many actin structures are localized to different parts of the cell, they are rarely independent and their dynamics often influence each other. In this review, we will discuss the dynamic reorganization of actin inside the cell and explore the crosstalk between different architectures.

## Actin structures in the cell: formation, architecture, and functions

**Figure 1.**
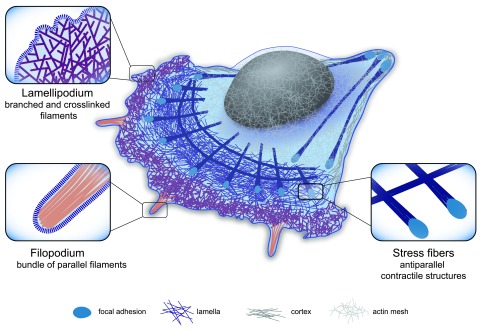
Cellular actin organization. Schematic representation of the three main actin structures found in the cell: 1. Lamellipodium: dense, branched network involved in cell protrusion. 2. Filopodium: a finger-like structure located at the leading edge of the motile cell composed of aligned filaments. Filopodia sense the extracellular environment and influence the direction of cell motility. 3. Contractile structure: dynamic structure made of antiparallel and/or mixed-polarity actin filaments associated with myosin. These structures play an important role in mechanical responses, providing force generation for different cellular functions. Zoomed regions highlight the specific actin organization of the different cellular actin structures.

The cell cytoplasm provides a large reservoir of actin monomers, and this reservoir is necessary for the assembly of complex actin-based structures ^4^. The initial step in building such a large structural array containing different types of actin filament arrangements ( Figure 1) requires controlled actin assembly and the inhibition of spontaneous polymerization ^4^. Two actin-binding proteins play a major role in regulating this process: thymosin and profilin ^5^. Thymosins sequester actin monomers to which they bind and thus fully block filament assembly ^6^. Profilins also bind to actin monomers but only inhibit spontaneous nucleation ^7^. Indeed the profilin/actin complex can add on to any pre-existing free filament barbed end and therefore participate in actin elongation ^8^.

Several types of proteins, classified as actin nucleators, can counteract the inhibitory effects on actin assembly by thymosin, profilin, or other monomer binding proteins ^9, 10^. These actin nucleators include the actin-related protein 2/3 (Arp2/3) complex, formins, and proteins containing WASP homology 2 (WH2) domains. However, the Arp2/3 complex ^11, 12^ is the only definitive actin nucleator, in the sense that it can overcome the limiting step in the formation of an efficient actin nucleus during assembly. Indeed, this complex contains two Arps, Arp2 and Arp3, that mimic an actin dimer ^11^. Other actin nucleators, including formins ^13, 14^ or WH2-domain containing proteins ^15^, appear to stabilize pre-existing dimers rather than generating or mimicking new ones ^16, 17^. Interestingly, profilin in yeast and mammalian cells can inhibit Arp2/3 complex nucleation activity, thus favoring the actin filament elongation activity of formin or Ena/VASP ^18, 19^.

The lamellipodium is a dense branched array of filaments that occurs at the leading edge of a motile cell and its formation is dictated by the activity of the Arp2/3 complex ( Figure 1 and 20). This specific type of actin organization pushes forward the plasma membrane during motility ^1, 21, 22^. This property relates to the lamellipodium’s optimal composition of arrays of growing actin filaments, which are oriented at ±35° with respect to the membrane. Once growing actin filaments extend beyond ~1 µm, they form parallel filament bundles and emerge as finger-like protrusions called filopodia ^23, 24^.

Filopodia direct how the cell probes the extracellular matrix (ECM) ( Figure 1 and 25) and control the orientation of lamellipodium ^26^. The parallel filament bundles within the filopodium also serve as tracks for protein transport ^27^. Filopodia are ~1–10 µm long ^28, 29^, with 10–30 actin filaments crosslinked in parallel arrays by fascin ^30^. Structural models predict that the densely packed nature of these actin arrays is important for the filaments to resist the loads coming from the membrane, such that filament elongation (by insertion of monomeric actin at the growing tip) remains uninhibited ^29, 31^. Moreover, the filaments within the filopodium have a turnover rate of ~20 mins ^32^ and hence are far more stable than those filaments within the lamellipodium, which have a turnover rate of ~1 min ^33^, or even only a few seconds at the very front of the lamellipodium ^34^.

The cell can also contain actin structures assembled from short filaments that are the sites for the action of molecular motors of the myosin family. Depending on their orientation, the short filaments can act as tracks for myosin or as contractile fibers, such as the transverse arcs or ventral stress fibers ^35^, and the perinuclear actin cap ( Figure 1 and 36). Radial and ventral stress fibers, oriented parallel to the migration axis ^37^, are anchored at focal adhesions at one (radial) or both (ventral) ends ^35^. Transverse arcs are formed just behind the lamellipodium ^35, 38^. Ventral stress fibers are made of filaments of >2 µm in length, whereas transverse fibers are made of shorter filaments of ~1 µm in length. These fibers contain on average 10–30 filaments by width section ^39^. Filament polarities inside stress fibers can be random (i.e. mixed polarity), graded, or sarcomeric (i.e. anti-parallel) ^39, 40^. Contractility is triggered by myosins that mediate sliding of anti-parallel filaments along each other ^41^. The equilibrium between contractile stress and adhesion strength can act as a modulator of cellular tension ^42^ and of the conversion of mechanical signals (tension) into biochemical signals (focal adhesion maturation), thus regulating the communication between the cell and the ECM ^36, 43^. Indeed, the assembly of stress fibers may only occur once the cell is under mechanical stress ^36^. Ventral fibers allow the retraction of the motile cell’s trailing edge ^39^ and may also initiate cell motility ^44^. By connecting the lamellipodium and the lamella, the transverse arcs, in the flattened perinuclear region ^45^, participate in the persistence of cell motility ^35, 46^. The perinuclear cap, a structure consisting of actomyosin fibers positioned around the nucleus, regulates the shape and position of the nucleus ^47^.

**Figure 2.**
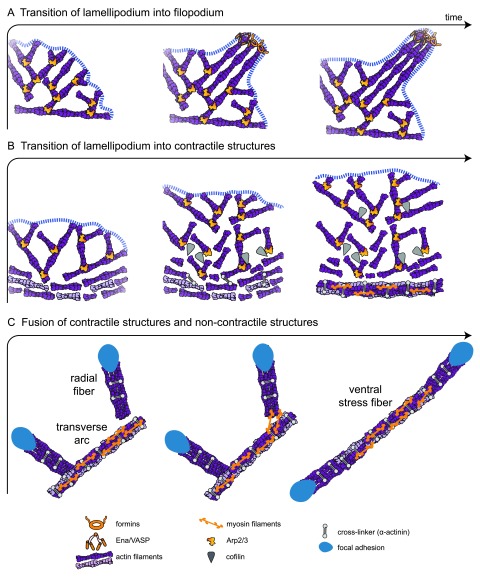
Actin architecture transitions. ( **A**) In the convergent elongation model, the transition from lamellipodium to filopodium involves the formation of parallel actin filaments from a branched network created by the Arp2/3 complex. Elongation factors, like Ena/VASP or formin, protect barbed ends from capping protein and induce the rapid polymerization of parallel bundles. ( **B**) The transition from lamellipodium to contractile structures is triggered by the disassembly of the branched network at the rear of the lamellipodium by ADF/cofilin and myosin. Myosin induces actin filament alignment and the formation of fibers stabilized by crosslinkers, such as α-actinin. ( **C**) The fusion of contractile structures (the transverse arcs) and non-contractile structures (the radial fibers) can lead to the formation of ventral stress fibers. In this scheme, myosins connect a transverse arc and two radial fibers and, after contraction, align the radial fibers with the transverse arc, creating a ventral stress fiber. This contractile antiparallel fiber is anchored at its two ends to focal adhesions.

The actin structures described above are highly dynamic in terms of formation, elongation/contraction, and disassembly, and these processes can be interdependent ( Figure 2). Therefore, to have a more complete understanding of cellular actin organization, it is essential to take into account the cytoskeleton dynamics inside the cell.

## From one actin structure to another: dynamical transitions

### From lamellipodium to filopodia

The potential for filopodia to emerge from the lamellipodium near the plasma membrane ( Figure 2A) raised the question of how a structure made of parallel actin bundles can arise from a densely branched actin network. Two overlapping theoretical models have attempted to explain this transition: the convergent elongation model and the nucleation model ^23, 48, 49^.

According to the convergent elongation model, filopodia are initiated by the reorganization of the branched actin network due to a fine-tuning of actin filament elongation at their growing ends ^25^. The branched actin filaments of the lamellipodium are short due to the regulation of their growth by capping proteins ^50, 51^. An attractive hypothesis to explain the transition between short filaments in the lamellipodium and the longer filaments driving filopodium formation is that some of the barbed actin filament ends in the lamellipodium are protected from capping proteins by cellular elongation factors such as Ena/VASP proteins ^52, 53^ or formins ^54^ and will therefore grow longer. In support of this hypothesis, Ena/VASP and formin proteins have been observed at filopodia tips ^30, 55^ and can induce filopodia formation ^56, 57^.

Moreover, depletion of capping protein promotes filopodia formation at the expense of lamellipodium extension, and Ena/VASP proteins have been shown to play an important role in filopodia formation ^58^. Indeed, Ena/VASP proteins promote the convergence of filament barbed ends and have an enhanced activity when bound to trailing barbed ends in a fascin bundle, thus allowing the trailing ends to catch up with the leading barbed ends ^59^. Longer actin filaments can, after positional fluctuations and bending, be captured and aligned into bundles by fascin ( Figure 2), depending on the angle of their association ^60, 61^. These initial thin bundles can be further reinforced by other actin filaments to form a rigid body that is necessary for filopodium growth ^29^. Convergence of actin network filaments into filopodia-like bundles can be recapitulated by both *in vitro* reconstitution ^23, 62^ and Monte Carlo simulation ^63^.

The nucleation model is supported by the observation that filopodia can form even when the lamellipodium is absent as a consequence of lack of the Arp2/3 complex or its activation ^64– 67^. In this model, formin and/or Ena/VASP promoting *de novo* tip nucleation form actin filaments in filopodium. Further support for this model comes from the recent observation that fibroblasts lacking Arp2/3 complex produce more prominent filopodia than wild-type cells ^68^. However, it is not yet clear how precisely filaments are initiated in the absence of the Arp2/3 complex. In a very elegant study using fission yeast, inhibition of the Arp2/3 complex disturbed the balance of different actin structures that were, in effect, competing for actin monomers from the same reservoir and resulted in the enhanced formation of formin-dependent structures ^69^. The abundance of filopodia when the Arp2/3 complex is knocked down might also be explained by the disruption of actin homeostasis causing an increased incidence of spontaneous assembly of actin filaments in the cytoplasm ^68^. A proportion of these spontaneously formed actin filaments may be capped by Ena/VASP and/or formins, whose activities are enhanced by the absence of the Arp2/3 complex, to promote filopodia formation ^49^. Together, the two models are not necessarily mutually exclusive and might be reconciled by a capture elongation model mediated by Ena/VASP or formins.

**Figure 3.**
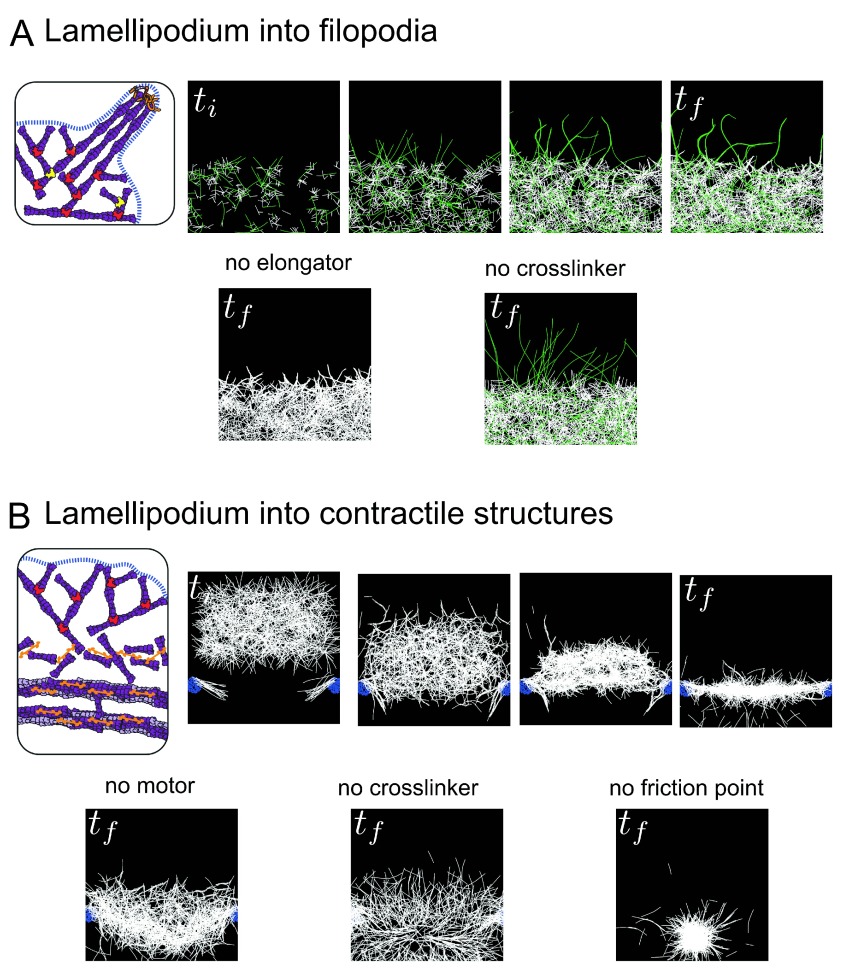
Simulation of transitions between actin structures. ( **A**) Emergence of filopodia-like protrusions from a lamellipodium-like network. Simulations were performed using the Cytosim software. In the top panel, the actin network grows by branched nucleation via the Arp2/3 complex, and a proportion of actin filaments grow longer due to capture of their growing barbed ends by an elongation factor (formin/VASP, green filaments). Actin filaments contact each other by chance due to thermal fluctuations and are stabilized in bundles by crosslinkers (fascin). In the bottom panel, the presence of elongation factors in the simulation is essential for the emergence of protrusions (left), while the crosslinkers are necessary to group the protrusions into one rigid bundle (right). ( **B**) Transition between lamellipodium-like and stress fiber-like networks. Simulations were performed using the Cytosim software. In the top panel, a branched network is formed and moved towards friction points (mimicking focal adhesions nucleating dorsal fibers) associated with parallel filaments. In the contact zone, the action of crosslinkers and myosins induces the disassembly of the branched network leading to the formation of a contractile structure of anti-parallel filaments. This structure is further compacted by a slow vertical flow (~centripetal actin flow) until it co-aligns with the friction points to form one contractile fiber. In the bottom panel, in the absence of motors, the network has no tension and is thus highly curved and spread (left). The crosslinkers are essential to maintain the connectivity between the filaments and form a continuous actin structure (middle). The friction points are essential to keep the network elongated at a given length, otherwise the network collapses to one point in the middle due to the tension (right). *t _i_* and *t _f_* indicate initial time and final time of simulations (empirical).

To interrogate and illustrate the dynamic transition from lamellipodium to filopodia, we performed mathematical simulations using the cytoskeleton simulation software Cytosim ^70^. In the simulation, a lamellipodium-like branched network was grown by distributing the Arp2/3 complex-like nucleators within a broad, two-dimensional area ( Figure 3A, top and 61). To create the variation of lengths among those actin filaments, formin-like (could also be Ena/VASP-like) entities were added to capture the barbed ends of growing actin filaments and accelerate filament elongation. The growing actin filaments then extended out of the lamellipodium network and merged into bundled filaments by fascin-like crosslinkers. In the simulation, a synergy between the modulation of actin filament elongation at growing barbed ends by Ena/VASP and/or formin and actin filament crosslinking into tight bundles is sufficient ( Figure 3A, top) and necessary to induce filopodium formation ( Figure 3A, bottom panel).

Although the principle of the transition from lamellipodium to filopodia may be simple, it is quite difficult to identify exactly which proteins or pathways are involved in the formation of a filopodium. There exists potential competition or redundancy between different cellular actors, illustrated by formins that constitute a large family of different isoforms ^71^. Moreover, the interactions between filaments and the membrane could also regulate this transition. The tension produced by the membrane can determine filopodia dimensions ^29^ and can induce filament alignment in protrusions, even in the absence of crosslinkers ^72^.

### From lamellipodium to contractile structures

A considerable amount of information about the assembly mechanisms of contractile structures has been obtained from numerous studies using live cell imaging ^36, 38, 46, 73, 74^. The current model for the assembly of radial fibers is based on a simple mechanism of initiation, whereby the fibers are generated by formin-mediated nucleation at focal adhesions ^75^. Following this initiation step, the growing actin filaments are brushed into parallel bundles by the retrograde flow toward the cell center ^38^. Radial fibers further recruit crosslinked filaments from the lamella, giving rise to an organization of filaments with graded polarity ^36^.

The model for the formation of transverse arcs is clearly different to that of radial fibers ^38^. Transverse arcs are assembled by the end-to-end annealing of myosin filaments and actin bundles that have come from the reorganization of the Arp2/3 complex branched network at the back of the lamellipodium ( Figure 2 and 46). The reorganization of a branched network into actin bundles of mixed polarity includes several steps. First, disassembly factors such as ADF/cofilin and glia maturation factor (GMF) disconnect the network by debranching the Arp2/3 complex links ^76– 78^. Second, the released short filaments are captured by myosin and actin filament crosslinkers such as α-actinin, which are present in the lamella to trigger the formation of small bundles ( Figure 2B). Third, the alignment of the filaments is enforced by the high mechanical stress produced by the interaction between focal adhesions and the ECM, and by centripetal flow at the lamellipodium/lamella interface ^79^. Fourth, the nascent bundles are pushed away from the cell edge by the actin centripetal flow ^74^, while condensing and forming transverse arcs, until they encounter focal adhesions and pre-formed radial fibers ^38, 80^. The radial fibers and transverse arcs will then associate with crosslinked actin bundles incorporating into the ends of radial fibers through the activity of myosin filaments.

We have also performed simulations of the transition from a branched actin network to a contractile fiber using Cytosim and the same simulation starting point using Arp2/3 complex-like nucleators as described above ( Figure 3A). To mimic focal adhesions nucleating the radial fibers, two small zones of adherence (friction points) with a few parallel filaments growing toward the cell center were placed at the bottom of a growing branched network. Arp2/3 complex connections were removed to simulate the lamellipodium debranching effect mediated by ADF/cofilin or GMF, and then motors (to simulate myosins) and crosslinkers (to simulate α-actinin) were added. A slow, directed flow was added to simulate the effect of centripetal actin flow. With only these few ingredients, the transition from a branched, non-contractile network to a mixed polarity, contractile fiber emerged from numerical simulations ( Figure 3B, top panel). These ingredients all seem essential, since removal of the motors, crosslinkers, or friction points all prevented the efficient formation of the contractile cable ( Figure 3B, bottom panel).

## Other transitions

### Fusion of contractile and non-contractile structures

Radial fibers can associate with transverse arcs by the incorporation of myosin II filaments and subsequently develop into ventral fibers ^37, 38^. During this process, first, two independent radial fibers connect with a pre-existing transverse arc that is pushed to the trailing edge of the motile cell by the flow. As a consequence, arc contractile forces get transmitted to radial fibers. Second, the distal parts of the transverse arc dissociate because of local stress relaxation ( Figure 2C). Finally, the radial fibers fuse with what remains of the contracting transverse arc to form a ventral stress fiber that is attached to focal adhesions at both ends ^38^.

In addition, a ventral stress fiber could be formed by the fusion of two dorsal stress fibers, without transverse arc incorporation ^80^. This latter case has been observed in Arp2/3 complex knockdown cells ^38^.

### Filopodia disassembly and their fate

The mechanisms by which filopodia disassemble remain to be determined. However, stationary filopodia can be disassembled into small bundles by ADF/cofilin ^81^. Filopodia may also develop kinks after a decrease and/or change of direction of the actin flow between the lamellipodium and lamella leading to their integration into the lamella ^73, 74, 82^. In both scenarios, short actin bundles generated by filopodia disassembly would then participate in the formation of contractile structures, by feeding pre-existing contractile fibers with actin filaments.

## Conclusion

Cellular functions depend on complex actin choreography. To orchestrate such a diversity of actin organizations, the dynamic integration of different mechanistic pathways is necessary. Some pathways are quite specific to the formation and maintenance of a particular basic actin organization, but because these actin structures may reorganize and transform, they may also indirectly participate in the emergence of other structures. The prevalence of these different basic actin organizations also varies in different cell types (e.g. lamellipodia predominate over filopodia in keratocytes and neutrophils, whereas filopodia predominate over lamellipodia in dendritic cells or neuronal growth cones). Within a given cell type, the predominance and/or existence of the different actin structures can be regulated to achieve specific functions, for example during collective migration ^83^. Thus, focusing on the behavior of a single type of actin structure may only provide an incomplete view of its formation and maintenance *in vivo*. Hence, the development of more appropriate experimental systems that can reconstitute more than one actin structure at a time should improve the understanding of the complexity of cellular actin dynamics. Mathematical simulations demonstrate that only a few components and simple boundary conditions are sufficient to mediate transitions between or during the emergence of complex actin structures. These mathematical approaches may also help in elaborating more appropriate experimental systems to unveil the general laws behind dynamic cytoskeletal reorganization.

## Supplemental movie legends

Movie S1 (related to Figure 3A)

Transition of lamellipodium into filopodia: assembly of parallel bundles (filopodia) triggered by the dense branched network (lamellipodium).

Movie S2 (related to Figure 3B)

Transition of lamellipodium into contractile structures. Formation of a contractile structure generated by the combined action of 3 parameters: actin flow, molecular motors and crosslinkers.
